# Changing Parental Attitudes Towards Rotavirus Vaccine

**DOI:** 10.7759/cureus.35348

**Published:** 2023-02-23

**Authors:** Zuhal Gundogdu, Ozge Yendur Sezer

**Affiliations:** 1 Pediatrics, Kocaeli University, Child Health and Diseases, Umuttepe Campus, Kocaeli, TUR

**Keywords:** rotavirus vaccine, information, cost, parental attitudes, parental belief

## Abstract

Background: Rotavirus is known to be one of the most common infections, usually associated with severe diarrhea. Despite the existence of two licensed vaccines, many countries, including Turkey, have not included rotavirus vaccination in their nationally funded vaccination program. This article explores what factors influence parents' decisions about whether to have their children vaccinated against rotavirus and which factors changed from 2010 through 2016.

Materials and Methods: Data were collected over two periods via questionnaires. The first period was from January 2009 through March 2010, and data were gathered from a semi-private pediatric outpatient clinic in Kocaeli, Turkey. The second period was from August 2015 through May 2016, and data were collected from parents during their pediatric outpatient clinic visits.

Two questionnaires were designed to find out the rotavirus vaccination status of the children, socio-demographic factors, and reasons for excluding/accepting the rotavirus vaccine. The level of knowledge about the rotavirus vaccine was investigated. Parents indicated their level of agreement with each statement using a five-point Likert scale.

Results: While only 3.8% of the parents accepted the rotavirus in 2009-2010, it increased to 69.5% in 2015-2016. Significant factors influencing parents’ decision to vaccinate their children for rotavirus were advice from a pediatrician, a lack of correct and timely rotavirus information, and the cost of the vaccine.

Conclusions: The acceptance of the rotavirus vaccine depends on parental perceptions, which may be influenced by accurate and timely information, the advice of their healthcare provider, and inclusion in the nationally funded vaccination program. In contrast to other studies reported, the education level of the mothers and fathers and their job types appear to be important. It was also found that parents’ attitudes and perceptions changed over time.

## Introduction

The most common cause of severe diarrhea in young children around the world is rotavirus infection. According to statistics, rotavirus-related infections cause an average of 1600 child deaths annually, making them the second most common cause of mortality in children under five years old that can be prevented by vaccination after pneumococcal pneumonia [1-6].

While rotavirus infection is the predominant cause of morbidity in developed countries, it is also a significant cause of mortality for children under the age of five in underdeveloped countries [1-6]. Rotavirus is responsible for 10%-20% of severe diarrhea cases and 25%-55% of cases that necessitate hospital admission globally [3-5]. More than 80% of rotavirus-related deaths take place in underdeveloped nations, typically in Southeast Asia and Africa [3-6].

RotaTeq (Merck and Co., PA, USA) and Rotarix (GSK Biologicals, Rixensart, Belgium) are two efficient rotavirus vaccines that have been licensed since 2006 and are advised for use in all countries by the WHO, particularly in those with high diarrhea-related mortality in children under the age of five [1].

The introduction of efficient and accessible rotavirus vaccines could significantly reduce the number of fatalities worldwide caused by diarrhea. The WHO broadened its recommendation for rotavirus vaccination use in 2009 to cover all nations, with a focus on those with high rates of death from diarrhea. To yet, however, the rotavirus vaccination has mostly only been distributed in nations with low rates of death due to diarrhea [5]. In high- and middle-income nations that have thus far used rotavirus vaccines, significant decreases in morbidity and death owing to rotavirus and diarrhea have been seen [7].

The effectiveness and reliability of rotavirus vaccination have been evaluated in a double-blinded, placebo-controlled, phase III study in 63,225 babies (31,675 in the vaccine group, 31,552 in the placebo group) from 11 South American countries [7]. It was found that the rotavirus vaccine was effective in preventing diarrhea and decreased the incidence of rotavirus gastroenteritis as well as hospital admissions [7]. In April 2009, the World Health Organization Strategic Advisory Group of Experts (SAGE) recommended that all national immunization schedules include rotavirus vaccination for infants [8].

Rotavirus infections occur in Turkey throughout the year; however, they are usually more common between September and May [9]. Although infections do vary with respect to regions, a recent study reported rotavirus frequency to be between 7.7% and 73.7% [9]. The death rate due to diarrhea in Turkey decreased, following the widespread application of oral fluid treatment. However, baby deaths may still occur due to diarrhea-related complications.

The Ministry of Health decides whether to add a vaccine to the national immunization program after consulting with an advisory board [10]. G1P(8) (54%), G2P(4) (12%), G3P(8) (3%), G4P(8) (9%), and, in recent years, G9P(8) and G9P(6) (both 4%), are the serotypes of the most common rotaviruses [11]. The most common serotypes seen in Turkey are G1-G4 and, increasingly in recent years, G9 [12]. However, in another study, it is reported that a large number of genotypes were observed, including common, uncommon, and mixed types, indicating a marked heterogeneity of rotavirus strains circulating in Turkey with major differences in the normally reported prevalences of the common genotypes, such that the prevalence of G3 and G1 was increased and that of G9 and G2 decreased from 2014 to 2016 [13-14].

Neither of the two types of commercially available rotavirus vaccines is government-funded in the Turkish routine vaccination program [10]. The rotavirus vaccine was only available and generally recommended, in private clinics for a fee. While some parents choose to vaccinate their children at their own expense, others receive partial reimbursement from private health insurance providers [10]. 

This study aimed to explore the changing attitudes of parents and parental characteristics over an approximately six-year period and what factors influenced their decision whether to have their children vaccinated against rotavirus based on the health belief model [15]. Fundamental components of the health belief model are perceived benefits, harm, susceptibility, severity, self-efficacy, and cues to action. All these components are usually evaluated simultaneously [16].

## Materials and methods

Two time periods were used to conduct this study. First, survey information was gathered from 262 parents in a semi-private pediatric outpatient clinic in Kocaeli, Turkey, between January 2009 and March 2010. A survey was once again used to collect the second set of data, this time from 302 parents, who responded to it while visiting the Kocaeli University Hospital's pediatric outpatient clinic during a routine visit between August 2015 and May 2016. They were healthy children between the ages of 0 and 18 years old. Before participating in the survey, all parents gave their informed consent. This study was approved by the Kocaeli University Ethical Committee (KOU KAEK 2015/242). Data were collected from parents who brought their children to the outpatient clinic for routine checks. They were approached at random, and once they gave their consent, the study was conducted face-to-face. Parents of the children with immune deficiencies, chronic diseases, or who were born prematurely or at a small for gestational age (SGA) were excluded from the study.

The questionnaire was created to investigate the rotavirus status of children and the socio-demographic characteristics of the families, including childbirth date and gender, family income, parents' level of education, parents' ages, and the number of other children they have. It also investigated the location of the residential address (urban/inner-city, suburban, or rural), the parents' line of work, and the parent's reasons for not vaccinating their children against rotavirus. The survey's content was based on the health belief statements for the rotavirus vaccine adapted from Taylor and Newman (2000) [15]. Using a five-point Likert scale, parents expressed their level of agreement with each statement, with options ranging from "strongly agree" to "strongly disagree." The responses were transformed into an ordinal scale with scores ranging from 1 to 5, with one indicating strong disagreement and five indicating strong agreement. In both studies, the parents were also asked about their reasons for accepting or refusing the vaccine. They were also questioned about their level of knowledge about rotavirus and its source.

Statistical analysis was conducted using SPSS, version 17 (IBM Inc., Armonk, NY, USA). The normal distribution test was evaluated with the Kolmogorov-Smirnov Test. Numerical variables with normal distribution were given as mean ± standard deviation, numerical variables not showing normal distribution as median (25th-75th percentiles), and categorical variables as frequency (%). Differences between groups were tested with the Mann-Whitney U test and Kruskal Wallis one way variance analysis and Dunn's multiple comparison test for numerical variables that do not have a normal distribution. Relationships between variables were determined by Spearman Correlation Analysis. For the testing of two-sided hypotheses, p < 0.05 was considered sufficient for statistical significance.

## Results

For the study conducted in 2009-2010, 67.6% (n = 177) of the questionnaires were filled out by mothers only, 28.6% (n = 75) were completed by fathers and mothers together, and 3.1% (n = 8) were completed by fathers alone. The remaining (n=2) 0.8% were completed by other family members present. In 2015-2016, 80.1% (n = 242) of questionnaires were filled out by mothers only, and 19.9% (n = 60) were completed by fathers only. The socio-demographic characteristics of the families participating in 2009-2010 and 2015-2016 are compared, and only the mothers' jobs were comparable as shown in Table 1.

**Table 1:** Basic population-related characteristics of the parents.

Demographic factors	(N=262) (2009-2010)	(N=302) (2015-2016)
Income	p<0.001	
1 (low-income)	18 (6.9%)	6 (2%)
2 (middle-income)	100 (38.2%)	86 (28.5%)
3 (upper middle income)	107 (40.8%)	188 (62.3%)
4 (high-income)	37 (14.1%)	22(7.3%)
Father's education	p<0.001	
1 (primary school)	60 (22.9%)	0
2 (secondary school)	130 (49.6%)	80 (26.5%)
3 (college)	23 (8.9%)	148 (49%)
4 (university)	49 (18.7%)	74 (24.5%)
Mother's education	p<0.001	
1 (primary school)	110 (42.5%)	4(1.3%)
2 (secondary school)	99 (38.2%)	124(41.1%)
3 (college)	16 (6.1%)	110(36.4%)
4 (university)	37 (14.1%)	64(21.2)
Number of children	p<0.001	
1	145 (55.6%)	79 (26.2%)
2	90 (34.3%)	152 (50.5%)
>=3	27(10.3%)	71 (23.5%)
Mother's job	p=0.615	
1 (professional/managerial)	10 (3.8%)	19 (6.3%)
2 (skilled)	29 (11.1%)	23(7.7%)
3 (semi-skilled)	11 (4.2%)	21 (7.1%)
4 (manual)	6 (2.3%)	6 (2.0%)
5 (other)	206 (79.5%)	233 (77.2%)
Father's job	p<0.001	
1 (professional/managerial)	46 (17.8%)	23 (7.6%)
2 (skilled)	58 (22.1%)	28 (9.3%)
3 (semi-skilled)	27 (10.4%)	46 (15.2%)
4 (manual)	127 (48.5%)	195 (64.6%)
5 (other)	4 (1.5%)	10 (3.3%)
Father's age (in years)	p<0.001	
18-24	5 (1.9%)	0
25-34	155 (59.6%)	115 (38.1%)
35-44	96 (36.7%)	159 (53%)
45-54	6 (2.3%)	28 (9.3%)
Mother's age (in years)	p<0.001	
18-24	45 (17.2%)	14 (4.6%)
25-34	181 (69.6%)	162 (53.6%)
35-44	35 (13.4%)	112 (37.1%)
45-54	1 (0.4%)	14 (4.6%)
Location of residential address	p=0.001	
Urban/inner city (1)	187 (71.4%)	135 (44.7%)
Suburban (2)	41 (15.6%)	157 (52%)
Rural (3)	34 (13.5%)	10 (3.3%)

While only 3.8% of parents accepted rotavirus in 2009, 69.5% of parents accepted the vaccine in 2015-2016. It may be that parental education and professions, as well as parental income, in 2015-2016 were higher than those in 2009-2010. Parental age is also older in 2015-2016 than in 2009-2010. The number of children in the family is higher in 2015-2016 than it was in 2009-2010, too. Results on whether the number of children in the family had any effect or not on vaccination rates need to be evaluated further.

Parents believe that getting their children vaccinated by all the recommended vaccines is important since it has a high median score of 4 agreed or strongly agreed in 2015-2016 but in 2009-2010 parents’ opinion about rotavirus vaccine were not clear as median score is 3 shown in Table 2. In 2015-2016, parents generally believed that their child had a high chance of being infected with rotavirus if not immunized and also appeared to believe that the infection was more serious and that the possible negative repercussions of rotavirus vaccination were less than in 2009-2010.

**Table 2:** Parental health beliefs regarding the rotavirus vaccine from Kocaeli, Turkey, between 2009 and 2016. Data are given as mean (SD) and median scores for the responses measured on a five-point Likert scale.

Statements	N=262; Mean (SD) Median score (Between 2009 and 2010)	N=302; Mean (SD) Median score (Between 2015 and 2016)
The vaccine is effective in preventing rotavirus diarrhea	3.45 (1.04) 3	3.92 (0.87) 4
The vaccine is worthwhile if the only benefit is preventing complications in 1–2 of 1000 children with rotavirus diarrhea	3.10 (0.89) 3	3.86 (0.95) 4
The risks of the rotavirus vaccine outweigh the benefits	3.15 (0.88) 3	3.43 (1.24) 4
The vaccine is worthwhile if the only benefit is preventing the discomfort of rotavirus diarrhea	3.21 (0.83) 3	3.98 (0.92) 4
Getting all immunizations is important to my child's health	4.18 (1.00) 4	3.74 (1.14) 4
The child is likely to get rotavirus diarrhea if not immunized	3.24 (0.97) 3	3.65 (1.03) 4
The rotavirus vaccine is unnecessary because rotavirus diarrhea is a minor illness	3.11 (0.97) 3	3.84 (1.17) 4
The vaccine is worthwhile if the only benefit is preventing time lost from work	3.23 (1.04) 3	3.74 (1.03) 4
The vaccine is worthwhile even if immunity is not lifelong	3.32 (0.71) 3	3.86 (0.96) 4
I am uncomfortable with the number of shots my child receives	2.47 (0.95) 2	3.01 (1.39) 3

In a separate study carried out in 2009 comparing parents’ perceptions of the seriousness of various diseases, responses to "This vaccine is unneeded because this disease is a minor illness" for rotavirus in 2009-2010 were evaluated in order to gauge parents' perceptions of the seriousness of the disease or the significance of the rotavirus vaccine. It was discovered that the parents in 2009-2010 neither agreed or disagreed with the seriousness of the rotavirus (50.6%), and therefore had no strong opinion about rotavirus infection [17].

The "Risks of rotavirus vaccine outweigh benefits" statement looked into the worry about the side effects of the vaccine. Because the rotavirus vaccination can cause nausea, fever, and diarrhea, parents were reluctant to ask for it. While 118 parents, or 40%, disagreed with the statement regarding side effect hesitancy, 115 parents, or 38%, neither agreed nor disagreed, and 69 parents, or 22%, disagreed with the statement regarding the alleged side effects of the rotavirus vaccine in 2015-2016. Regarding the last statement, "I am uncomfortable with the number of shots my child receives," 30% (n = 90) of parents disagreed, 35% (n = 106 neither disagreed nor agreed, and 34% (n = 105) agreed with the statement in 2015-2016 (Table 2).

**Figure 1:**
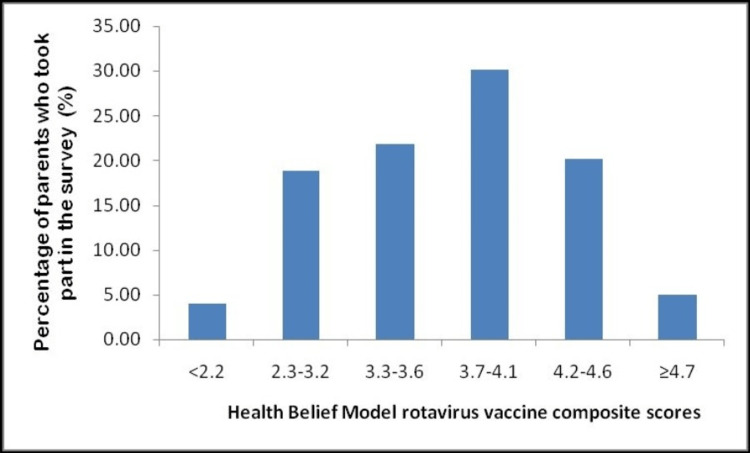
Distribution of composite scores among parents who completed the survey in 2015-2016.

The composite score is calculated as the ratio of the sum of the highest responses to the number of questions, as previously described in Ref. [15]. It basically represents the parents' composite health belief score and percentage. Using each parent's survey responses, the composite health belief score was calculated by dividing the sum of individual statement scores by the number of statements for which the parent indicated a level of agreement as shown in Figure 1.

Each parent was also asked their reasoning if their response to the rotavirus vaccine was "Yes." Parents were more positively influenced, especially in 2015-2016, as shown in Table 3. Not having enough information and financial concerns, such as not being able to afford the vaccine, swayed parents’ decisions. Parents' perceptions of the importance of rotavirus vaccination increased from 2010 to 2016, probably because of better and more specific information.

**Table 3:** Responses to the question “Is rotavirus vaccines needed for your child?” in Kocaeli, Turkey between 2009-2010 and 2015-2016.

		2009-2010 n (%)	2015-2016 n (%)
If no, which reasons		252 (96.1%)	92 (30.4%)
	Already had the infection	3 (1.1%)	5 (1.6%)
	Vaccine price	104 (39.6%)	10 (3.3%)
	Not enough time	4 (1.5 %)	1 (0.3%)
	No knowledge	123 (46.9%)	69 (22.8%)
	Scared of adverse effects	12 (4.5%)	1 (0.3%)
	Insufficient information about vaccines	4 (1.5%)	6 (1.9%)
If yes, which reasons		10 (3.8%)	210 (69.5%)
	Positively influenced	4 (1.5%)	87 (28.8%)
	Prevent the disease and complications	2 (0.7%)	99 (32.7%)
	Positively influenced and to prevent the disease and its complications	4 (1.5%)	24 (7.9%)

Table 4 shows the level and source of information about rotavirus vaccination. While the majority of parents had never heard about the rotavirus vaccine previously only 20.9% (n=63) of parents felt they had enough detailed knowledge about the rotavirus vaccine. The effect of the average monthly income of the parents who took part in the survey with respect to vaccination rates. The low income of families was less in 2015-2016 than in 2009-2010. Also, upper middle-income of families were more in 2015-2016 than in 2009-2010.

**Table 4:** Source/level of knowledge about rotavirus vaccine.

Source/level of knowledge about rotavirus vaccination	Different sources	Media	Friends/Family	Health professionals	Their own family physician	Enough and accurate information from a pediatrician
	184 (60.9%)	16 (5.3%)	18 (6.0%)	68 (22.8%)	15 (5.0%)	63 (20.9%)

At the end of the survey, each parent in the pediatric outpatient clinic in 2015-2016 was given detailed information on the rotavirus vaccine. After being fully and properly informed about rotavirus infection, the severity of the disease, and the availability of vaccines, parents' vaccine acceptance rates increased dramatically (p < 0.05) from 69.5% to 88.0% (n = 266).

## Discussion

These findings describe a change in parents’ attitude about the rotavirus vaccine over an approximately six-year period. While only 10 (3.8%) out of 262 parents accepted against rotavirus vaccine in 2009-2010, 210 (69.5%) parents accepted it in 2015-2016. There was no strong opinion towards rotavirus vaccination in 2009 when parents also reflected that they were not willing to have rotavirus vaccination for their children even if it was funded and included in the national vaccination program [17]. A similar attitude is again reflected in 2016; however, this view changed dramatically after parents were given information on the effects and the burden of the disease after they filled out the survey.

Additionally, when compared to parents with lesser levels of education, there was a statistically significant difference in vaccination intention, according to a finding similarly published by MacDougall et al. [18]. Given that parents with greater levels of education were much more inclined to have their children receive vaccinations, the mothers' and fathers' levels of education and their occupations are key factors. The amount of parental knowledge, parental wealth, access to vaccines, the accuracy of vaccine information, and the education level of the mothers all appear to have played a significant role in immunization acceptance [19-20]. One should also note that some of the socio-demographic factors are also different and better in the 2015-2016 study, such as income level, parents’ education, and especially fathers’ jobs, etc. Nevertheless, the education levels of the mothers and fathers and their job types appear to be important, as these parents were significantly willing to have their children immunized. The significance of social media should also be emphasized in a when looking at the characteristics of parental vaccine refusal. 

Vaccine costs would mostly impact those in the low socioeconomic levels, as people in the middle and higher-income groups would be able to afford the vaccine [18]. The cost-effectiveness of a new health intervention is one of several crucial factors considered by decision-makers before an intervention is introduced [21-22]. Our study found that both the cost and a lack of high-quality information influenced parental decisions about rotavirus vaccines.

Objective and accurate information given to parents by healthcare workers, particularly by pediatricians, seemed to have an effect on parents’ changing their decisions. Similar results were reported by Le Ngoc Tho et al. [23] where 93.7% of parents were positive for vaccination after being fully informed, and in another study, this rate was 90% [24].

It is clear that parents are positively influenced by the advice of doctors, especially that of pediatricians [10, 17]. However, lack of awareness and knowledge of the potential health burden of rotavirus among parents is not taken seriously by health care providers or family physicians [20]. This was also determined in the present study. A number of parents replied that their doctors told them that not completing the vaccination course would be sufficient. A similar result was found by Bedford and Lansley (2006), who reported that, apart from providing the parents with information, the attitude and approach of doctors or health care providers were also important factors in accepting a vaccine [25]. This indicates that providing accurate and timely information on immunization issues to pediatricians, and probably other physicians and healthcare workers responsible for the health care for children is important.

 In an internet-based study carried out in Germany with 6025 participants [26], 95% of the participants reported that the most important source of advice on vaccines is advice from pediatricians, the same view endorsed by the present study and others [10, 17]. The majority of participants expressed a positive experience with immunizations in their children and relied on their pediatricians as the major source of information on this subject. Participants, who also mentioned books and the internet as information sources were less satisfied once they had been informed by their pediatricians. This indicated that providing accurate and timely information on immunization issues to pediatricians, and probably other physicians and health care workers responsible for the health care for children, as well as guidance to relevant internet sources would be important for providing objective information to parents [26]. In our view, providing information about vaccinations during pre-natal maternal education sessions may play a key role, a view also put forward by Hu et al. (2017) [27] where they also suggest that a strong partnership should be established between obstetricians and pediatricians or other vaccine related healthcare workers.

Furthermore, parents’ views and perceptions towards all vaccines are not equal, as some conditions are considered more serious than others [10, 14]. For this reason, when giving advice to parents, healthcare professionals should consider the parents’ cognitive processes as well as the benefits of vaccination, and the seriousness and threat of the diseases. It would probably be advisable to re-educate healthcare providers and physicians and provide them with more support before starting a vaccine campaign, as they play a key role in the public acceptance of new vaccines [28]. In the absence of public endorsement by the government and the implementation of public health education programs not only for parents but also for healthcare workers, parents did not rate a disease such as a rotavirus infection as an important health concern. This was also the case with nurses, who would recommend the rotavirus vaccine if there was a national recommendation and the vaccine was publicly funded, as previously reported [16]. In this study, 20% (n = 60) of parents thought rotavirus illness was not a very serious disease and vaccination was therefore not necessary, 32% (n = 99) were not sure, and 49% (n = 148) thought rotavirus infection was serious enough to warrant rotavirus vaccination.

One of the other main parental barriers to vaccination was the confusion and difficulty in tracking vaccination schedules. In addition, parents cited a lack of awareness regarding the importance of vaccines, missing due dates, and fear of the possible complications and side effects of vaccines as reasons for not completing vaccination. As a result, it is critical to remind and reassure parents about vaccine efficacy and safety. Given the widespread use of mobile phones, the use of Android and iOS apps designed for vaccination reminders can be helpful. Existing apps have been reviewed, and a new app design was suggested by Abahussin and Albarrak [29].

According to McIntosh et al. (2016), tracking the degree and type of vaccine reluctance is necessary since these variables may change over time. Measuring vaccine hesitancy is also essential for the proper development of measures to increase vaccine coverage and for monitoring. This paper also documents the evolution of parental attitudes over time. Pediatricians may have a significant impact on parental vaccine decisions, and vaccine hesitation may be exclusive to certain vaccines but not all [30].

To lessen the pervasive impacts, vaccine hesitancy and refusal should be regularly observed, researched from medical, psychological, social, political, and ethical perspectives, and appropriately addressed [30]. In this study, the views and actions of parents concerning the rotavirus vaccine between 2010 and 2016 were compared. Parents in 2009 did not strongly agree or disagree with the seriousness of the rotavirus, hence there was no strong sentiment in favor of the vaccine. But in 2016, there was more understanding and acceptance. These could serve as a good guide for those making decisions on whether to add rotavirus vaccination to the national immunization program.

## Conclusions

The acceptability of the rotavirus vaccine depends on a number of factors, although greater information, especially from a pediatrician, and government funding for the vaccine may sway parents' opinions. It would be crucial to include rotavirus vaccinations in the standard schedule for childhood immunization to prevent the childhood mortality brought on by rotavirus and deaths from diarrheal illnesses. The attainment of these significant and shared objectives might be greatly helped by increased efforts to make these vaccines available to all children.

## References

[1] Tate EJ, Burton AH, BoschiPinto C (2012). 2008 estimate of worldwide rotavirus-associated mortality in children younger than 5 years before the introduction of universal rotavirus vaccination programmes: a systematic review and meta-analysis. Lancet Infect Dis.

[2] Pittet LF, Posfay-Barbe KM (2012). Pneumococcal vaccines for children: a global public health priority. Clin Microbiol Infect.

[3] Pickering LK, Cleary TG (2004). Infections of the gastrointestinal tract. Krugman’s Infectious Diseases of Children.

[4] Steele AD, Madhi SA, Cunliffe NA (2016). Incidence of rotavirus gastroenteritis by age in African, Asian and European children: relevance for timing of rotavirus vaccination. Hum Vaccin Immunother.

[5] Tate JE, Burton AH, Boschi-Pinto C, Parashar UD (2016). Global, regional, and national estimates of rotavirus mortality in children >5 years of age, 2000-2013. Clin Infect Dis.

[6] Du Y, Chen C, Zhang X (2022). Global burden and trends of rotavirus infection-associated deaths from 1990 to 2019: an observational trend study. Virol J.

[7] Ruiz-Palacios GM, Pérez-Schael I, Velázquez FR (2006). Safety and efficacy of an attenuated vaccine against severe rotavirus gastroenteritis. N Engl J Med.

[8] Karafillakis E, Hassounah S, Atchison C (2015). Effectiveness and impact of rotavirus vaccines in Europe, 2006-2014. Vaccine.

[9] Alkan S, Dindar Demiray EK, Akça A, Önder T, Vurucu S. (2022). Nozokomiyal rotavirüs enfeksiyonları. BSJ Health Sci.

[10] Gundogdu Z, Gundogdu O (2011). Parental attitudes and varicella vaccine in Kocaeli, Turkey. Prev Med.

[11] Staat MA, Bernstein DI (2014). Vaccine. Feigin and Cherry's Textbook of Pediatric Infectious Diseases.

[12] Durmaz R, Kalaycioglu AT, Acar S (2014). Prevalence of rotavirus genotypes in children younger than 5 years of age before the introduction of a universal rotavirus vaccination program: report of rotavirus surveillance in Turkey. PLoS One.

[13] Durmaz R, Bakkaloglu Z, Unaldi O (2018). Prevalence and diversity of rotavirus A genotypes cirulating in Turkey during a 2-year sentinel surveillance period, 2014-2016. J Med Virol.

[14] Tapisiz A, Bedir Demirdag T, Cura Yayla BC (2019). Rotavirus infections in children in Turkey: a systematic review. Rev Med Virol.

[15] Taylor JA, Newman RD (2000). Parental attitudes toward varicella vaccination. The Puget Sound Pediatric Research Network. Arch Pediatr Adolesc Med.

[16] Glanz K, Rimer BK, Viswanath K (2015). Health Behavior and Health Education: Theory, Research, and Practice. Health Behavior and Health Education. Theory, Research and Practice.

[17] Gundogdu Z (2020). Parental attitudes and perceptions towards vaccines. Cureus.

[18] MacDougall DM, Halperin BA, Langley JM, MacKinnon-Cameron D, Li L, Halperin SA (2016). Knowledge, attitudes, beliefs, and behaviors of parents and healthcare providers before and after implementation of a universal rotavirus vaccination program. Vaccine.

[19] Luies SK, Hossain MT, Sarma H (2019). Awareness among parents about pneumococcal conjugate vaccine in routine immunization program to prevent pneumococcal pneumonia in Bangladesh. Cureus.

[20] Seale H, Sitaresmi MN, Atthobari J (2015). Knowledge and attitudes towards rotavirus diarrhea and the vaccine amongst healthcare providers in Yogyakarta Indonesia. BMC Health Serv Res.

[21] Sigei C, Odaga J, Mvundura M, Madrid Y, Clark AD (2015). Cost-effectiveness of rotavirus vaccination in Kenya and Uganda. Vaccine.

[22] Fishbein DB, Broder KR, Markowitz L, Messonnier N (2008). New, and some not-so-new, vaccines for adolescents and diseases they prevent. Pediatrics.

[23] Le Ngoc Tho S, Ader F, Ferry T (2015). Vaccination against serogroup B Neisseria meningitidis: perceptions and attitudes of parents. Vaccine.

[24] Gauthier A, Jauffret-Roustide M, Jestin C (2008). Nicolle 2006 survey: knowledge, attitudes and behaviours against the risk of infection.

[25] Bedford H, Lansley M (2006). Information on childhood immunisation: parents’ views. Commun Pract.

[26] Heininger U (2006). An internet-based survey on parental attitudes towards immunization. Vaccine.

[27] Hu Y, Chen Y, Wang Y, Song Q, Li Q (2017). Prenatal vaccination education intervention improves both the mothers' knowledge and children's vaccination coverage: evidence from randomized controlled trial from eastern China. Hum Vaccin Immunother.

[28] Morin A, Lemaître T, Farrands A, Carrier N, Gagneur A (2012). Maternal knowledge, attitudes and beliefs regarding gastroenteritis and rotavirus vaccine before implementing vaccination program: which key messages in light of a new immunization program?. Vaccine.

[29] Abahussin AA, Albarrak AI (2016). Vaccination adherence: review and proposed model. J Infect Public Health.

[30] McIntosh ED, Janda J, Ehrich JH, Pettoello-Mantovani M, Somekh E (2016). Vaccine hesitancy and refusal. J Pediatr.

